# Predictive model using systemic inflammation markers to assess neoadjuvant chemotherapy efficacy in breast cancer

**DOI:** 10.3389/fonc.2025.1552802

**Published:** 2025-03-24

**Authors:** Yulu Sun, Yinan Guan, Hao Yu, Yin Zhang, Jinqiu Tao, Weijie Zhang, Yongzhong Yao

**Affiliations:** Division of Breast Surgery, Department of General Surgery, Nanjing Drum Tower Hospital, The Affiliated Hospital of Nanjing University Medical School, Nanjing, China

**Keywords:** breast cancer, systemic inflammation markers, pCR, prognosis, predictive model

## Abstract

**Background:**

Pathological complete response (pCR) is an important indicator for evaluating the efficacy of neoadjuvant chemotherapy (NAC) in breast cancer. The role of systemic inflammation markers in predicting pCR and the long-term prognosis of breast cancer patients undergoing NAC remains controversial. The purpose of this study was to explore the potential predictive and prognostic value of systemic inflammation markers (NLR, PLR, LMR, NMR) and clinicopathological characteristics in breast cancer patients receiving NAC and construct a pCR prediction model based on these indicators.

**Methods:**

A retrospective analysis was conducted on 209 breast cancer patients who received NAC at Nanjing Drum Tower Hospital between January 2010 and March 2020. Independent sample t-tests, chi-square tests, and logistic regression models were used to evaluate the correlation between clinicopathological data, systemic inflammation markers, and pCR. Receiver operating characteristic (ROC) curves were utilized to determine the optimal cut-off values for NLR, PLR, and LMR. Survival analysis was performed using the Kaplan-Meier method and log-rank test. A predictive model for pCR was constructed using machine learning algorithms.

**Results:**

Among the 209 breast cancer patients, 29 achieved pCR. During a median follow-up of 68 months, 74 patients experienced local or distant metastasis, and 56 patients died. Univariate logistic regression analysis showed that lymph node status, HER-2 status, NLR, PLR, and LMR were associated with pCR. ROC curve analysis revealed that the optimal cut-off values for NLR, PLR, and LMR were 1.525, 113.620, and 6.225, respectively. Multivariate logistic regression analysis indicated that lymph node status, NLR, and LMR were independent predictive factors for pCR. Survival analysis demonstrated that lymph node status, NLR, and LMR were associated with prognosis. Machine learning algorithm analysis identified the random forest (RF) model as the optimal predictive model for pCR.

**Conclusion:**

This study demonstrated that lymph node status, NLR, and LMR had significant value in predicting pCR and prognosis in breast cancer patients. The RF model provides a simple and cost-effective tool for pCR prediction, offering strong support for clinical decision-making in breast cancer treatment and aiding in the optimization of individualized treatment strategies.

## 1 Introduction

Female breast cancer has been the leading cause of global cancer incidence nowadays which seriously does harm to physical and mental health of woman (1, 2). Nowadays, neoadjuvant chemotherapy (NAC) is a standard therapeutic strategy for some kinds of breast cancer, which can promote improved breast conservation therapy rates and provide timely and individualized information on chemotherapy sensitivity (3). Pathological complete response (pCR) following neoadjuvant therapy serves as a key measure of treatment effectiveness. It is commonly defined as the complete absence of invasive cancer cells in the surgical specimen, including both the primary tumor site and lymph nodes (4, 5). Research has demonstrated that patients achieving pCR tend to have more favorable long-term outcomes, such as significantly prolonged disease-free survival (DFS) and overall survival (OS) (6–8). As a result, the role of pCR as a surrogate endpoint for clinical benefit for NAC has been widely acknowledged.

However, since not all patients achieve pCR, it is essential to identify predictive biomarkers for treatment response to guide the development of individualized treatment strategies. Therefore, many studies are trying to explore tumor biomarkers for breast cancer prognosis, but due to economic and technical limitations, most of them remain in the laboratory stage and have not been applied to clinical large-scale (9–12). It is hoped that a precise, comprehensive, and easily accessible biomarker can be identified as soon as possible to predict the efficacy of NAC and the prognosis of breast cancer patients.

Inflammation predisposes to the development of cancer and promotes all stages of tumorigenesis such as initiation, promotion, invasion, and metastasis (13, 14). Systemic and local tumor inflammation all play an important role in the pathophysiology of breast cancer. Among the various systemic inflammation markers, the neutrophil-to-lymphocyte ratio (NLR), platelet-to-lymphocyte ratio (PLR), and lymphocyte-to-monocyte ratio (LMR), neutrophil-to-monocyte ratio (NMR) have attracted significant attention due to their ability to reflect the balance between pro-tumor inflammatory responses and anti-tumor immune activity (15–17). In addition, peripheral blood testing is widely used in clinical practice, which is convenient, cheap and fast to operate. Therefore, indicators of peripheral blood inflammation are expected to become reliable biomarkers for predicting pCR and long-term prognosis.

However, the predictive and prognostic value of different systemic inflammation markers in breast cancer remains controversial. Based on this, this study aimed to comprehensively investigate the relationship between peripheral blood inflammation markers (such as NLR, PLR, LMR and NMR) and the efficacy of NAC or prognosis in breast cancer patients. By collecting peripheral blood data and relevant clinicopathological information from breast cancer patients, this study analyzed the applicability of these markers as tools for predicting short-term treatment outcomes and long-term survival rates in breast cancer. In addition, this study utilized machine learning algorithms to construct various pCR prediction models based on several blood inflammation markers. Through analysis and comparison, the optimal model was ultimately identified to predict the efficacy of NAC in different breast cancer patients. Therefore, this study not only developed a simple and cost-effective pCR prediction model based on systemic inflammatory markers but also demonstrated its broad potential for clinical application. The model supports precision medicine by evaluating NAC efficacy and optimizing personalized treatment plans.

## 2 Methods

### 2.1 Patients

Female patients with invasive breast cancer between January 2010 and March 2020 at Nanjing Drum Tower Hospital were retrospectively screened. The inclusion criteria were as follows: (1) patients aged ≥18 years; (2) patients pathologically diagnosed with invasive breast cancer using hollow needle biopsy before NAC; (3) patients who underwent NAC prior to surgery; (4) patients with available complete medical data. The exclusion criteria were as follows: (1) patients with metastatic breast cancer or a history of other primary malignant tumors (2) patients who received other types of neoadjuvant therapy, including radiotherapy or endocrine therapy; (3) patients with any chronic inflammatory diseases, including autoimmune diseases, such as systemic lupus erythematosus, rheumatoid arthritis, Sjögren’s syndrome, etc; (4) Patients lost to follow-up. This study was approved by the Ethics Committee of Nanjing Drum Tower Hospital and conducted in compliance with the Declaration of Helsinki.

### 2.2 Clinicopathologic analysis

Patients information were collected from the electronic medical records of Nanjing Drum Tower Hospital, including complete blood count results within one week before NAC, clinicopathological characteristics, and treatment plans. Neutrophil-to-lymphocyte ratio (NLR) = neutrophil count (10^9^/L)/lymphocyte count (10^9^/L). Platelet-to-lymphocyte ratio (PLR) = platelet count (10^9^/L)/lymphocyte count (10^9^/L). Lymphocyte-to-monocyte ratio (LMR) = lymphocyte count (10^9^/L)/monocyte count (10^9^/L). Neutrophil-to-monocyte ratio (NMR) = neutrophil count (10^9^/L)/monocyte count (10^9^/L) (18).

### 2.3 Outcomes and patients follow-up

The primary endpoint of this study was pCR. pCR was defined as the absence of invasive disease in the breast and axilla (ypT0ypN0 or ypTisypN0) (19). The secondary endpoint of this study were disease free survival (DFS) and overall survival (OS). DFS was calculated as the time (in months) from the date of diagnosis to the date of relapse (local, loco-regional or distant recurrence) or death. OS was calculated as the time (in months) from the date of diagnosis to the date of death due to any cause or the final follow-up time. Follow-ups were conducted every 3 months during the first 2 years after surgery, every 6 months for 3-5 years, and annually thereafter.

### 2.4 Statistical analysis

Statistical analyses were performed using SPSS 22.0 software. Continuous variables with a normal distribution, were analyzed using an independent samples t-test. Categorical variables were analyzed using the chi-square test. Univariate and multivariate logistic regression models were used to evaluate the correlation between clinicopathological characteristics and pCR. Receiver operating characteristic (ROC) analysis was used to evaluate the optimal cut-off value of systemic inflammatory markers. Survival analysis was conducted using the Kaplan-Meier method and log-rank test. A p-value of less than 0.05 was considered statistically significant.

### 2.5 The predictive model construction method

The pCR predictive model was constructed using the Support Vector Machine (SVM), Random Forest (RF), and K-Nearest Neighbors (KNN) algorithms sequentially through the PyCharm Professional 2023.1.3 software. Standard Deviation (SD), Root Mean Square Error (RMSE), and Correlation Coefficient (r) were used as evaluation metrics to determine the optimal model.

#### 2.5.1 Algorithm introduction

(1) SVM (Support Vector Machine)

SVM is a supervised algorithm used for both classification and regression tasks (20). Its goal is to identify the hyperplane that best separates classes in a high-dimensional space by maximizing the margin (distance) between the hyperplane and the closest data points from each class, called support vectors. The optimization can be expressed as:


$$min\frac{1}{2}{\left\|\omega \right\|}^{2}$$

subject to:


$$y_{i}\left(\omega \cdot x_{i}+b\right)\geq 1,\forall i$$

where ω is the weight vector, b is the bias, x_i_ represents the input features, and y_i_ is the class label (+1 or -1). For cases where the data is not linearly separable, SVM employs kernel functions (e.g., polynomial or RBF kernels) to project the data into a higher-dimensional space.

(2) RF (Random Forest)

RF is an ensemble method based on decision trees, commonly used for both classification and regression (21). It trains multiple decision trees on random subsets of the data and generates predictions by combining them-for classification via majority voting or for regression by averaging. Each tree is built using a bootstrap sample and splits nodes based on a random selection of features. The prediction formula is:


$$\hat{y}=\frac{1}{N}\sum_{i-1}^{N}T_{i}(x)$$

where T_i_(x) is the prediction from the i-th tree and N is the total number of trees. The randomization during training allows the algorithm to model diverse data patterns effectively while reducing overfitting and improving generalization.

(3) KNN (K-Nearest Neighbors)

KNN is a straightforward, non-parametric algorithm used for classification and regression (22). It assigns a class to a data point by finding its k-nearest neighbors in the feature space and determining the majority class among them or predicts a value by averaging their outputs. The similarity between data points is calculated using distance metrics, such as the Euclidean distance:


$$d(x,x^{\prime })=\sqrt{\sum_{i=1}^{n}(x_{i}-x_{i}^{'}{)}^{2}}$$

where x and x’ are feature vectors. KNN depends entirely on the training data, making it computationally expensive during prediction and sensitive to the value of k and the choice of distance metric.

Fine-tuning hyperparameters plays a crucial role in enhancing the effectiveness of machine learning models, as it significantly affects their performance, complexity, training processes, and generalization capabilities. This study examines the initial hyperparameter settings of various algorithms, as shown in **Table 1**.

**Table 1:** The initial setting of each algorithm’s hyperparameter.

Algorithm	Hyperparameter	Value Range
SVM	Kernel type	Linear, Polynomial, RBF, Sigmoid
	Regularization parameter C	0.1, 1, 10
	Gamma parameter	0.01, 0.1, 1
RF	Number of decision trees	100–500 (step size of 5)
	Maximum depth	1–91 (step size of 1)
	Maximum number of features	1–3
KNN	Number of neighbors (n_neighbors)	1–6 (step size of 1)
	Weights	‘distance’ or ‘uniform’

#### 2.5.2 Evaluation index

Different algorithms were used to evaluate the accuracy of the pCR prediction model. The evaluation metrics—standard deviation (SD), root mean square error (RMSE), and correlation coefficient (*r*)—provide a comprehensive assessment of the model’s performance. SD evaluates prediction consistency, RMSE measures accuracy by penalizing larger errors, and *r* assesses the linear relationship between predictions and actual outcomes. Together, these metrics ensure the model’s reliability in predicting pCR and supporting personalized clinical treatments. The calculations of evaluation indicators are as follows (23):


$$\text{SD}=\sqrt{\frac{1}{\text{N}}\sum_{\text{I}=1}^{\text{N}}{\left({\text{X}}_{\text{i}}-\bar{\text{X}}\right)}^{2}}$$

$$\text{RMSE}=\sqrt{\frac{\sum_{\text{i}=1}^{\text{n}}{\left({\text{y}}_{\text{i,actual}}-{\text{y}}_{\text{i,predicted}}\right)}^{2}}{\text{N}-1}}$$

$$\text{r}=\sqrt{1-\frac{\sum_{\text{i}=1}^{\text{n}}{\left({\text{y}}_{\text{i,actual}}-{\text{y}}_{\text{i,predicted}}\right)}^{2}}{\sum_{\text{i}=1}^{\text{n}}{\left({\text{y}}_{\text{i,actual}}-{\text{y}}_{\text{average}}\right)}^{2}}}$$

Where, y*_i_*_,actual_ represents the true value for the *i*-th sample, while y*_i_*_,predicted_ denotes its predicted value. y_average_ refers to the mean of the actual values, and *n* or *N* corresponds to the total number of samples. For the standard deviation formula, *x_i_* indicates the value of the *i*-th sample, $\overset{-}{\text{X}}$ represents the average of the sample values

## 3 Results

### 3.1 Patient characteristics

A total of 209 patients with breast cancer who received NAC were included in this research, with an average age of 50.9 ± 10.8 years (**Table 2**). 29 of them benefited from NAC and obtained pCR, while 180 unobtained pCR. There were no significant differences in age, menopausal status, and tumor size between the pCR group and the non-pCR group (P>0.05). Patients without lymph node metastasis were more likely to achieve pCR (P=0.008). In addition, The human epidermal growth factor receptor 2 (HER2)-positive subtype was also significantly increased in the pCR group (P=0.030), which may suggest that HER2 positivity is associated with a better pCR.

**Table 2:** Clinicopathological characteristics of breast cancer patients.

Characteristics	Total (n=209)	pCR (n=29)	Non-pCR (n=180)	P value
Age, years	50.9 ± 10.8	52.9 ± 9.4	50.6 ± 11.0	0.28
Menopausal state				
Premenopausal	97 (46.4)	11 (37.9)	86 (47.8)	0.324
Postmenopausal	112 (53.6)	18 (62.1)	94 (52.2)	
Tumor size				
≤ 2 cm	42 (20.1)	9 (31.0)	33 (18.3)	0.113
>2cm	167 (79.9)	20 (69.0)	147 (81.7)	
Lymph node metastasis				
Negative	58 (27.8)	14 (48.3)	44 (24.4)	**0.008**
Positive	151 (72.2)	15 (51.7)	136 (75.6)	
HER2 status				
Negative	118 (56.5)	11 (37.9)	107 (59.4)	**0.030**
Positive	91 (43.5)	18 (62.1)	73 (40.6)	
Molecular subtyping				
HER2	91 (43.5)	18 (62.1)	73 (40.6)	**0.032**
TNBC	46 (22.0)	7 (24.1)	39 (21.7)	
Luminal	72 (34.4)	4 (13.8)	68 (37.8)	

Presented as mean ± standard deviation or frequency (%).

HER2 human epidermal growth factor receptor 2; TNBC triple-negative breast cancer.

Bold values indicate that they are statistically significant at P<0.05.

### 3.2 Analysis of factors in relation to pCR in breast cancer patients

Univariate analysis manifested that lymph node metastasis (OR (95% CI): 0.347 (0.155-0.774), P=0.010), HER-2 status (OR (95% CI): 2.399 (1.070-5.375), P=0.034), NLR (OR (95% CI): 0.283 (0.130-0.617), P=0.001), PLR (OR (95% CI): 0.987 (0.975-0.998), P=0.019) and LMR (OR (95% CI): 1.214 (1.064-1.386), P=0.004) were the factors associated with the efficacy of NAC. The breast cancer group without lymph node metastasis, HER2-positive group, low NLR group, low PLR group, and high LMR group were more likely to achieve pCR (**Table 3**).

**Table 3:** Univariate logistic regression analysis of factors in relation to pCR in breast cancer patients.

Variable	OR value (95% CI)	P value
Lymph node metastasis	0.347 (0.155-0.774)	**0.010**
HER2 status	2.399 (1.070-5.375)	**0.034**
NLR	0.283 (0.130-0.617)	**0.001**
PLR	0.987 (0.975-0.998)	**0.019**
LMR	1.214 (1.064-1.386)	**0.004**
NMR	0.921 (0.817-1.037)	0.175

pCR, pathological complete response; HER2, human epidermal growth factor receptor 2; NLR, neutrophil-to-lymphocyte ratio; PLR, platelet-to-lymphocyte ratio; LMR, lymphocyte-to-monocyte ratio; NMR, neutrophil-to-monocyte ratio.

Bold values indicate that they are statistically significant at P<0.05.

Considering the lack of exact boundary values for NLR, PLR, and LMR in clinical practice, the optimal cut-off values for predicting pCR in this research were determined through Receiver Operating Characteristic (ROC) analysis, grouped by NLR (1.525), PLR (113.620), and LMR (6.225). The Area Under the Curve (AUC) values of them were 0.729, 0.640, and 0.685, respectively (**Table 4**).

**Table 4:** Optimal cut-off values of NLR, PLR, and MLR based on ROC curve analysis for prediction of pCR in breast cancer patients.

Index	AUC	95% CI	Cut-off value	P value
NLR	0.729	0.621-0.836	1.525	**<0.001**
PLR	0.640	0.537-0.742	113.620	**0.016**
LMR	0.685	0.576-0.794	6.225	**0.001**

NLR, neutrophil-to-lymphocyte ratio; PLR, platelet-to-lymphocyte ratio; LMR, lymphocyte-to-monocyte ratio; ROC, Receiver Operating Characteristic; pCR, pathological complete response; AUC, area under the curve. Bold values indicate that they are statistically significant at P<0.05.

Multivariate binary logistic regression was performed on the indicators with differences in univariate analysis. It was found that lymph node metastasis (OR (95% CI): 0.347 (0.140-0.862), P=0.023), NLR (OR (95% CI): 0.376 (0.143-0.990), P=0.048), and LMR (OR (95% CI): 2.828 (1.081-7.400), P=0.034) were factors associated with the efficacy of NAC (**Table 5**).

**Table 5:** Multivariate logistic regression analysis of factors in relation to pCR in breast cancer patients.

Variable	OR value (95% CI)	P value
Constant		0.018
Lymph node metastasis (- VS +)	0.347 (0.140-0.862)	**0.023**
HER2 status (- VS +)	2.155 (0.888-5.228)	0.090
NLR (≤1.525 VS >1.525)	0.376 (0.143-0.990)	**0.048**
PLR (≤113.620 VS >113.620)	0.747 (0.282-1.977)	0.557
LMR (≤6.225 VS >6.225)	2.828 (1.081-7.400)	**0.034**

pCR, pathological complete response; HER2, human epidermal growth factor receptor 2; NLR, neutrophil-to-lymphocyte ratio; PLR, platelet-to-lymphocyte ratio; LMR, lymphocyte-to-monocyte ratio.

Bold values indicate that they are statistically significant at P<0.05.

### 3.3 Survival analysis

**Figure 1:**
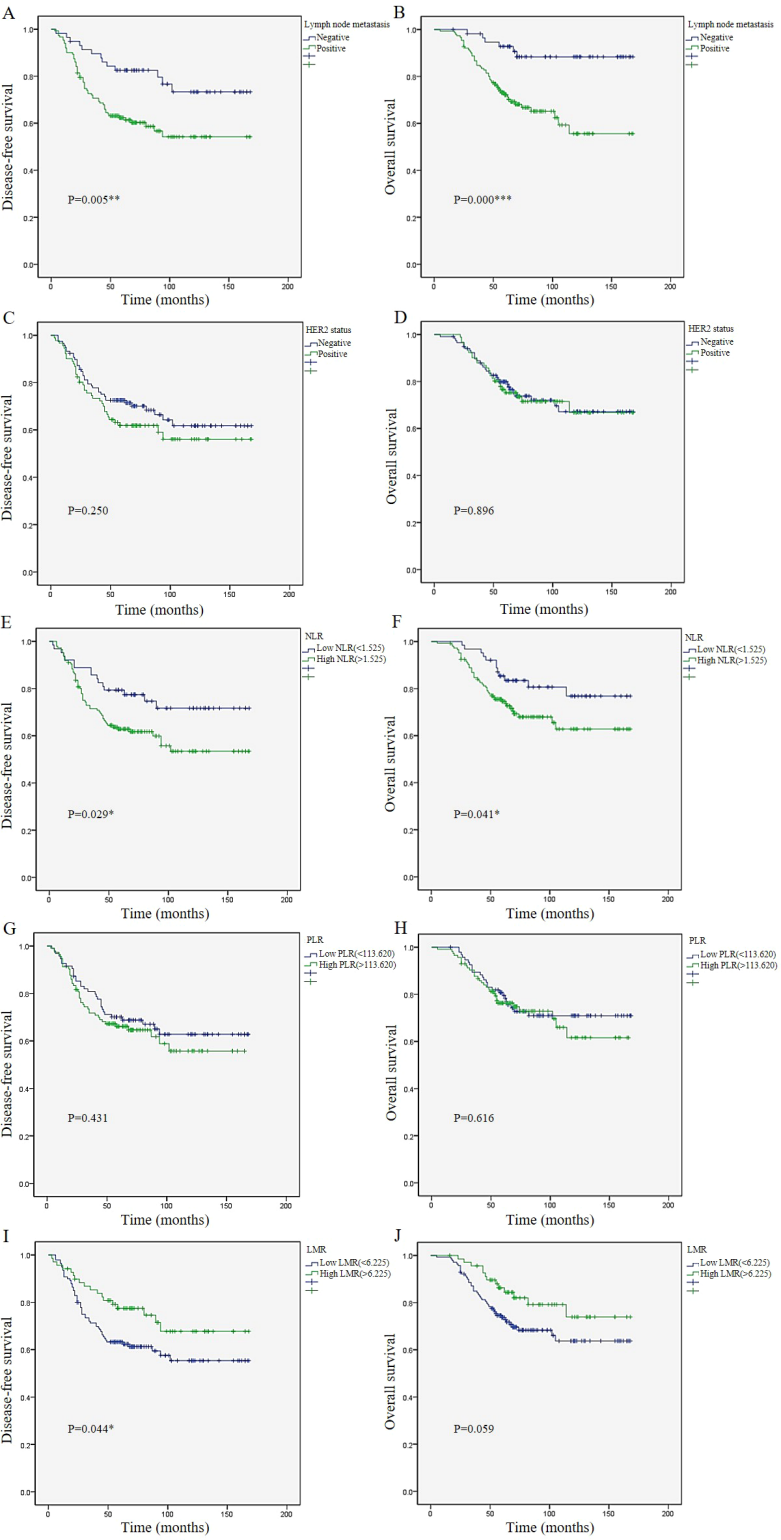
Survival analysis. Kaplan–Meier survival plots of lymph node metastasis **(A)**, HER2 status **(C)**, NLR **(E)**, PLR **(G)**, and LMR **(I)** for disease-free survival.Kaplan–Meier survival plots of lymph node metastasis **(B)**, HER2 status **(D)**, NLR **(F)**, PLR **(H)**, and LMR **(J)** for overall survival. p ≤ 0.05: * (statistically significant); p ≤ 0.01: ** (highly significant); p ≤ 0.001: *** (extremely significant).

During a median follow-up of 68 months (range: 5-168 months), 74 patients (35.4%) experienced local or distant metastasis, and 56 patients (26.8%) died. The average DFS was 115.7 months and the average OS was 130.3 months. Through Kaplan-Meier analysis (log-rank test), it could be observed that the absence of lymph node metastasis and lower NLR were associated with longer DFS (P=0.005, P=0.029) and longer OS (P<0.001, P=0.041). In addition, higher LMR was associated with longer DFS (P=0.044). Although it was not significantly associated with longer OS (P=0.059), a certain trend was observed. However, HER2 status and PLR were not associated with either DFS or OS (P>0.05) (**Figure 1**).

### 3.4 Performance analysis of different algorithm models

**Figure 2:**
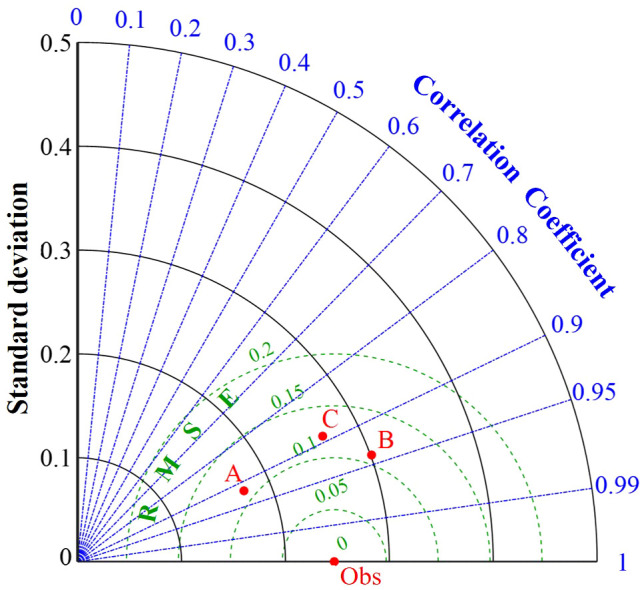
Performance comparison of different models. A, B and C represent pCR models constructed using SVM, RF and KNN algorithms respectively.

To further quantitatively describe the impact of Lymph Node Metastasis, NLR, and LMR on pCR, this study utilized machine learning algorithms to construct a pCR prediction model. The performance of different algorithm models were shown in **Table 6** and **Figure 2**. The performance of pCR predictive models constructed with different algorithms were described using the Taylor diagram. The Taylor diagram evaluated pCR predictive models from different algorithms by comparing predictions to observations using three metrics: SD, RMSE, and r. The radial distance indicated SD (larger values are farther from the center), the distance to Obs represented RMSE (shorter distances indicate higher accuracy), and the angle with the horizontal axis reflected r (smaller angles mean stronger correlations).

**Table 6:** Performance comparison of different predictive models.

Model algorithm	SD	RMSE	r
SVM	0.174	0.110	0.92
RF	0.301	0.109	0.94
KNN	0.265	0.121	0.89

SVM, support vector machine; RF, random forest; KNN, K-Nearest Neighbors; SD, standard deviation; RMSE, root mean square error; r, correlation coefficient.

The RF model performed the best (**Figure 2**). It had the lowest RMSE (0.109), indicating the smallest error, and the highest r (0.94), demonstrating the strongest fitting capability. Although its SD (0.301) was slightly higher than other algorithms, its overall predictive performance was superior to both SVM and KNN. SVM ranked second, with an RMSE of 0.110 and an r of 0.92, and it had the lowest variability (SD=0.174), making it the runner-up choice. KNN had the highest RMSE (0.121) and the lowest r (0.89), indicating the poorest performance (**Table 6**). In summary, RF was the optimal model. The optimal hyperparameter combination for the model algorithm through cross-validation and grid search was: n_estimators=200, max_depth=10, max_featurest= 2.

The RF algorithm outperforms SVM and KNN in pCR prediction due to its structural and topological advantages, which align with this study’s objectives (24). RF uses an ensemble of decision trees with bootstrap aggregating, reducing overfitting and improving robustness, making it effective for high-dimensional clinical data like inflammation markers and clinicopathological features. Its decision-tree framework automatically selects key features and captures complex nonlinear relationships between predictors and outcomes (25). This is critical for pCR prediction, where such factors interact intricately. In comparison, SVM is sensitive to parameter tuning, and KNN struggles with high-dimensional data due to its lack of modeling.

## 4 Discussion

This study retrospectively analyzed the predictive and prognostic value of several systemic inflammation markers in breast cancer patients receiving NAC and developed an optimal prediction model, aiming to explore whether these indicators could influence pCR and survival outcomes. We found that lymph node status, NLR, and LMR were significant predictors of pCR and were closely associated with recurrence and survival in breast cancer patients undergoing NAC. Therefore, we successfully developed an optimal model to predict pCR using these three factors with machine learning algorithms.

Inflammation plays a crucial role in the various stages of carcinogenesis and cancer treatment (13, 26). It not only promotes cancer initiation and progression by promoting tumor cell proliferation, invasion, and metastasis but also suppresses anti-tumor immune responses by altering the tumor microenvironment (27, 28). Neutrophils and monocytes can promote tumor angiogenesis and facilitate immune evasion by secreting pro-inflammatory factors and growth factors, while lymphocytes play a critical role in anti-tumor immunity (29, 30). Additionally, platelets contribute to tumor progression by supporting tumor cell proliferation and protecting them from immune system attacks (31, 32). Therefore, various inflammatory markers have been incorporated into clinical studies to assess systemic inflammatory status and immune function in patients. However, numerous studies have shown that the role of different systemic inflammatory markers in predicting treatment response and prognosis in breast cancer remains controversial.

NLR is one of the most extensively studied inflammatory markers. A meta-analysis by Cullinane et al. which included 8 studies with a total of 1,586 patients, demonstrated that a lower baseline NLR was associated with a higher pCR rate (OR (95% CI): 1.83 (1.15–2.91), P=0.0003) (33). Subsequently, another meta-analysis evaluating 17 studies showed that a high NLR was associated with a lower pCR rate (OR (95% CI): 1.620 (1.209–2.169), P<0.001) (34). Among this meta-analysis, 11 studies assessed the relationship between NLR and DFS in breast cancer patients, and indicated that elevated NLR was associated with poorer DFS (HR (95% CI): 2.269 (1.557–3.307), P<0.001). Furthermore, 6 studies revealed that elevated NLR was also associated with poorer OS (HR (95% CI): 1.691 (1.365–2.096), P < 0.001). These findings were entirely consistent with the results of our study, which showed that higher NLR values were associated with poorer pCR, DFS, and OS. Our study demonstrated that NLR was an independent predictor of pCR after NAC in breast cancer patients and a prognostic factor for DFS and OS. However, a small number of studies did not observe a significant predictive relationship between NLR levels and pCR and suggested that NLR was not an independent prognostic indicator for DFS (35, 36).

PLR, as a systemic inflammatory marker, remains controversial in its predictive and prognostic value for breast cancer patients undergoing NAC. A meta-analysis by Qi et al., which included 22 studies involving 5,533 patients treated with NAC, demonstrated that elevated PLR was associated with a lower pCR rate (HR (95% CI): 0.77 (0.67–0.88), P<0.001), poorer OS (HR (95% CI): 1.90 (1.39–2.59), P<0.001), and worse DFS (HR (95% CI): 1.97 (1.56–2.50), P<0.001) (37). Subsequently, another study also suggested that PLR was an independent predictor of pCR, which contradicted the findings of our study (38). However, two recent studies were fully consistent with the results of our study, indicating that high PLR groups showed lower pCR rates in univariate analysis, but PLR was not significantly associated with pCR in multivariate analysis and was unrelated to patient prognosis (39, 40). Interestingly, Dan et al. found that the difference in PLR before and after treatment, rather than pre-treatment or post-treatment PLR alone, was significantly associated with pCR (41).

Studies on LMR and NMR are relatively limited, and this study further enhances the understanding of these two inflammatory markers. Peng et al., through a retrospective analysis of 808 breast cancer patients undergoing NAC, found that pre-NAC LMR was an independent predictor of NAC efficacy (OR (95% CI): 1.771 (1.273–2.464), P=0.001) (42). This was consistent with our findings, as we observed that higher LMR was associated with better pCR rates and improved DFS. However, many studies suggested that higher LMR after NAC or before surgery was closely associated with higher pCR rates and better breast cancer prognosis (43, 44). This study did not find a relationship between NMR and NAC efficacy or patient prognosis, which was supported by some previous studies (42, 45). Therefore, this study suggested that NMR was unlikely to serve as an evaluation indicator for predicting NAC efficacy or survival prognosis.

A major highlight of this study was the identification of factors associated with pCR through multivariate analysis, followed by the construction of several pCR predictive models using machine learning algorithms and the selection of the optimal model to assist in guiding clinical decision-making. The higher accuracy of RF compared to SVM and KNN primarily stems from its algorithm structure and topology (21). In this study, the RF model achieved the smallest RMSE (0.109), and the highest r (0.94), indicating the lowest error and the strongest fitting ability. Therefore, RF outperformed SVM and KNN in terms of handling data complexity, robustness, and stability across different datasets, making it the optimal model in this study.

There are some limitations to this study. First, it is a single-center retrospective study. Future multi-center prospective trials and external validation could be conducted to make the model more universal and generalizable. Second, the study involves a relatively long enrollment period, during which breast cancer treatment methods have continuously evolved, and the emergence of targeted therapies has led to changes in NAC regimens. Finally, peripheral blood inflammation markers are influenced by many factors, but this study has excluded patients with inflammatory diseases or use of specific medications. For model algorithm optimization, future efforts will aim to expand the sample set and incorporate deep learning techniques, such as neural networks, to build pCR prediction models with greater adaptability and enhanced accuracy.

## 5 Conclusion

This study indicated that lymph node status, NLR, and LMR were independent predictors of pCR and were strongly associated with the prognosis of breast cancer patients undergoing NAC. To further quantitatively analyze the impact of these three indicators on pCR, pCR predictive models were constructed using machine learning algorithm. The RF prediction model could effectively and accurately evaluate the efficacy of NAC in breast cancer patients, providing a simple and cost-effective tool for personalized treatment strategies.

## Data Availability

The original contributions presented in the study are included in the article/supplementary material. Further inquiries can be directed to the corresponding author.
